# Genetic Characterization of Japanese Encephalitis Virus Isolates Circulating in Mosquitoes from Pig and Sheep Farms in Shanghai, China

**DOI:** 10.3390/ani14243653

**Published:** 2024-12-18

**Authors:** Hailong Zhang, Dan Li, Jiayang Zheng, Yan Zhang, Zongjie Li, Ke Liu, Beibei Li, Yafeng Qiu, Donghua Shao, Soesoe Wai, Jianchao Wei, Zhiyong Ma, Juxiang Liu

**Affiliations:** 1 College of Veterinary Medicine, Hebei Agricultural University, Baoding 071000, China; 2 Shanghai Veterinary Research Institute, Chinese Academy of Agricultural Sciences, Shanghai 200241, China; 3 Department of Veterinary Public Health, University of Veterinary Science, Yezin 15013, Myanmar

**Keywords:** Japanese encephalitis virus, *Culex tritaeniorhynchus*, *Anopheles sinensis*, phylogenetic analysis, Shanghai

## Abstract

Japanese encephalitis virus (JEV) is regarded as an emerging and reemerging pathogen that is a primary cause of viral encephalitis in humans. However, there is a scarcity of research on the prevalence of JEV genotypes across the different mosquito species in Shanghai. In this study, the diversity of mosquito species and prevalence of JEV in pig and sheep farms in Shanghai were surveyed in 2022. A total of 24,073 mosquitoes, belonging to four genera and seven species, were collected from pig and sheep enclosures in the Chongming, Jiading, Pudong, Fengxian, and Jinshan Districts of Shanghai. *Culex tritaeniorhynchus* was identified as the predominant species (87.09%, 20,965/24,073) with the highest JEV detection rate. Six strains of JEV were isolated in this study, and genetic analysis revealed that five strains (SH22-M5, SH22-M9, SH22-M14, SH22-M41, and SH22-M52) belonged to genotype I (GI), while one strain (SH22-M1) was classified as genotype III (GIII). The sequence homology was highest between SH22-M9 and SD-1 (99.87%) and between SH22-M14 and SD12 (99.53%). SH22-M5, SH22-M41, and SH22-M52 shared the highest sequence homology with the HEN07011 strain (99.73–99.93%). SH22-M1 was most closely related to SH18, with a sequence homology of 99.8%. Additionally, for the first time, the GI JEV strain was isolated from mosquitoes in sheep sties in this study. The findings highlight the necessity of enhancing the surveillance of JEV in pigs and other livestock farms, including sheep, as well as monitoring the mosquitoes present in these environments. It is recommended that livestock farming areas be kept separate from human habitation to reduce the risk of JEV infections in humans.

## Simple Summary

Japanese encephalitis virus (JEV) is a major cause of viral encephalitis in hu-mans, but research on its genotype prevalence in mosquitoes in Shanghai is limited. This study surveyed mosquito diversity and JEV prevalence in pig and sheep farms in Shanghai in 2022. A total of 24,073 mosquitoes from four genera and seven species were collected across five districts. *Culex tritaeniorhynchus* was the most common species (87.09%, 20,965/24,073) with the highest JEV detection rate. Six JEV strains were isolated, with five (SH22-M5, SH22-M9, SH22-M14, SH22-M41, and SH22-M52) classified as genotype I (GI) and one (SH22-M1) as genotype III (GIII). SH22-M9 and SH22-M14 showed high homology with SD-1 (99.87%) and SD12 (99.53%), respectively. SH22-M5, SH22-M41, and SH22-M52 shared 99.73–99.93% homology with the HEN07011 strain. SH22-M1 was most similar to SH18 (99.8%). This study also reports the first isolation of a GI JEV strain from mosquitoes in sheep farms. The findings underscore the need for enhanced JEV surveillance in livestock and mosquito monitoring to reduce the risk of human infections, recommending the separation of livestock and human habitation.

## 1. Introduction

Japanese encephalitis (JE) is a major zoonotic infectious disease caused by the Japanese encephalitis virus (JEV). The first strain of JEV, denoted as the Nakayama strain, was isolated in 1935 from the brain tissue of a fatal case [1,2]. Although several species of mammals and birds are susceptible to JEV, the infections are mostly asymptomatic [3]. The clinical symptoms of JEV are primarily observed in humans and horses; however, piglets infected with JEV develop non-suppurative encephalitis, while adult pigs may experience reproductive disorders. It is estimated that nearly 3 billion people worldwide are at risk of JEV infection, and approximately 67,900 cases of JE are reported annually [4,5]. The mortality rate due to JE ranges from 20% to 30% [6], and 30–50% of survivors are left with permanent sequelae, including severe speech impairments and neurological symptoms. Although JE is a vaccine-preventable disease, it is gradually spreading from Asia to Europe, Australia, and Africa.

JEV is a zoonotic flavivirus belonging to the Flavivirus genus within the Flaviviridae family, which comprises over 70 species, including the dengue virus, yellow fever virus, West Nile virus, Zika virus, and tick-borne encephalitis virus [7,8]. The JEV genome comprises a single-stranded, positive-sense RNA, which encodes a polyprotein that is subsequently cleaved into three structural proteins (C, prM, and E) and seven non-structural proteins (NS1, NS2A, NS2B, NS3, NS4A, NS4B, and NS5) [9]. Based on the nucleotide sequence of the gene encoding the E protein, JEV is phylogenetically classified into five genotypes (from I to V), and the most prevalent genotypes are genotype I (GI) and/or genotype III (GIII) in most Asian countries [10]. JEV is a zoonotic mosquito-borne virus that circulates in a zoonotic cycle between vertebrate hosts via arthropod vectors. The transmission cycle of JEV was elucidated as early as the 1950s, revealing that pigs and ardeid wading birds serve as amplifying hosts, while *Culex* spp. mosquitoes serve as the primary vector [11,12]. *C.* sp. mosquitoes are the primary vector species for the transmission of JEV. However, studies have confirmed that over 30 species of mosquitoes belonging to the *Aedes*, *Anopheles*, *Armigeres*, and *Mansonia* genera also serve as vectors of JEV, with varying susceptibility and competence [13]. Following JEV infection, pigs and ardeid wading birds develop a high level of viremia that is sufficient for infecting mosquitoes, and thus, they play key roles, as amplifying hosts, in maintaining the JEV transmission cycle [12].

Shanghai is located in Eastern China and has a tropical monsoon climate. It is an international metropolis with numerous nature reserves that serves as an important location on the East Asia–Australasia migratory route for birds, hosting a variety of migratory bird species. This makes it a potential high-risk area for the spread of mosquito-borne viruses [1,5]. Although the implementation of vaccination programs has significantly reduced the incidence of Japanese encephalitis (JE) in Shanghai, outbreaks of JE [14,15,16] and dengue [17], continue to occur in nearby areas. The range of mosquito-borne disease transmission may have changed due to climate change and urbanization. Therefore, this study assesses the mosquito species and the prevalence of mosquito-borne viruses (JEV) in the Shanghai region. Understanding the distribution of dominant mosquito species and the risk of virus carriage can enable early warning, facilitate the timely identification of potential virus transmission hotspots, and allow for rapid responses. In addition, it can help assess the effectiveness of existing vaccination strategies, provide data support for the development of new vaccines or the strengthening of vaccine coverage, and lay the foundation for preventing and controlling outbreaks of mosquito-borne diseases.

## 2. Materials and Methods

### 2.1. Sample Collection

Mosquito samples were collected between June and August 2022 from pigsties and sheep sties in the Chongming, Jiading, Pudong, Fengxian, and Jinshan Districts of Shanghai (Table 1). The samples were collected on a daily basis using ultraviolet (UV) light traps (Wuhan Lucky Star Environmental Protection Technology Co., Ltd., Wuhan, China), with four black UV traps installed in the respective pig and sheep enclosures. The traps were set up before sunset at 5:00 p.m., and mosquitoes were collected at 9:00 a.m. the following morning. The collected mosquitoes were then frozen at −20 °C for 40 min, placed on ice for morphological identification and classification, and the blood-containing mosquitoes and male mosquitoes were excluded. Mosquitoes containing blood were excluded to prevent contamination from the viruses contained in blood meal. A total of 50 mosquitoes were transferred to 2 mL centrifuge tubes, to which sterile steel beads and 1 mL of Dulbecco’s Modified Eagle’s Medium (DMEM) supplemented with 10% penicillin–streptomycin were added. The centrifuge tubes were subsequently placed in a shaker device and agitated for 5 min at a frequency of 50. After shaking, the centrifuge tubes were centrifuged at 13,000 rpm for 20 min at 4 °C, and the resulting supernatant was stored at −80 °C for further analysis.

### 2.2. Virus Isolation and Identification

Total RNA was extracted from the mosquito samples using Trizol reagent (10296010CN, Thermo Fisher Scientific, Waltham, MA, USA), and the cDNA was synthesized as per default protocols using SuperScript™ III First-Strand Synthesis SuperMix (18080400, Thermo Fisher Scientific, USA). The nucleotide sequence of the E gene of JEV was amplified using the JEV-475-F and JEV-475-R primers designed in our laboratory. The PCR-positive samples were sent to Shanghai Shenggong Biotechnology Co., Ltd. (Shanghai, China) for sequencing using the primers JEV-E-F1 and JEV-E-R1 (Table S1).

JEV was isolated as described previously described [18], and the PCR-positive samples were homogenized and filtered through a 0.22 μm filter membrane. The filtrate was subsequently inoculated onto a monolayer of BHK-21 cells; grown in DMEM; and supplemented with 2% fetal calf serum (Gibco, Waltham, MA, USA), 100 U/mL penicillin (Sigma, Kawasaki, Japan), and 100 mg/mL streptomycin (Sigma) solution [19]. The inoculated BHK-21 cells were then cultured at 37 °C in an atmosphere of 5% CO_2_ for 5–7 days and monitored for cytopathic effects (CPEs) every 12 h. Samples that exhibited no CPEs were subjected to blind passage for three to five generations [20]. The positive isolates included those that exhibited CPEs in three consecutive passages. The total nucleic acid was extracted from the supernatant of the inoculated BHK-21 cells that exhibited CPEs and was used for PCR analysis. All the experiments for viral isolation were performed in the Biosafety Level 2 cell culture laboratory established at Shanghai Veterinary Research Institute, Shanghai, China.

### 2.3. Minimum Infection Rate (MIR)

The MIR of JEV was calculated using the following formula: (number of pools that tested positive for JEV/total number of specimens tested) × 1000. The MIR was expressed as the number of mosquitoes that tested positive for JEV per 1000 tested and assumes that a positive pool contains only 1 infected mosquito [21].

### 2.4. Phylogenetic Analyses

The E gene nucleotide sequences of the E gene of 52 strains of JEV were retrieved from GenBank and subjected to multiple sequence alignment analysis. The selected strains had been isolated across various time periods and from different countries and diverse hosts. The 52 E gene reference sequences of JEV retrieved from GenBank (Table S2) were subjected to multiple sequence alignment and phylogenetic analyses using MEGA X software (Version 11.0.13). The phylogenetic trees were constructed using the neighbor-joining method, and the bootstrap values were derived from 1000 iterations. The evolutionary distances were computed using the maximum composite likelihood method [22,23,24].

## 3. Results

### 3.1. Distribution of Mosquitoes

A total of 24,073 female mosquitoes belonging to seven species and four genera were collected using UVT mosquito traps from sheep and pig enclosures in the Chongming, Jiading, Pudong, Fengxian, and Jinshan Districts of Shanghai. The 24,073 mosquitoes included four species of *Culex*, one species of *Anopheles*, one species of *Aedes*, and one species of *Armigeres*. *C. tritaeniorhynchus* was the dominant mosquito species (87.09%, 20,965/24,073), followed by *C. bitaeniorhynchus* (4.73%, 1139/24,073), *An. sinensis* (4.13%, 995/24,073), *Ae. albopictus* (3.54%, 853/24,073), *C. pipiens pallens* (0.33%, 79/24,073), *C. pipiens* quinquefasciatus (0.08%, 20/24,073), and *Ar. subalbatus* (0.09%, 22/24,073) (Table 2).

### 3.2. Detection and Isolation of JEV

**Figure 1:**
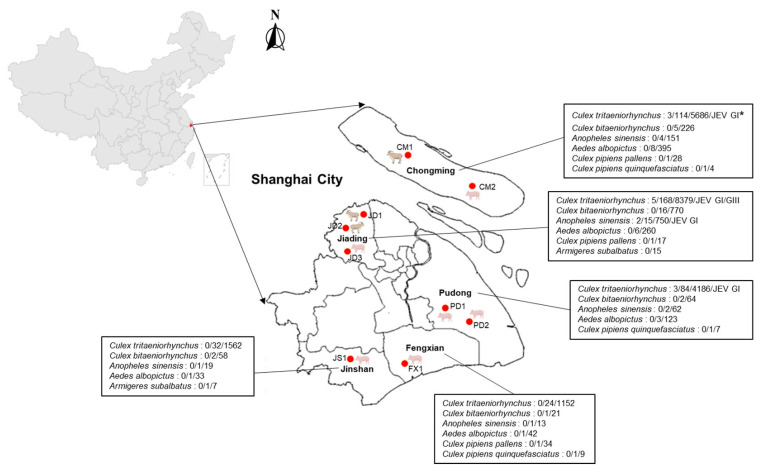
The counties of the mosquito sample collection site in Shanghai Province, China. Maps were created by an online map service system. Red dots are pig or sheep farms where mosquito samples were collected. *: JEV-positive pools/pools tested/number of mosquitoes/genotype.

The mosquitoes were sorted into 497 pools according to their species, location, and date of collection; each sample pool comprised 50 mosquitoes, with pools of fewer than 50 mosquitoes counting as one pool. A total of 45 of the 497 pools had fewer than 50 mosquitoes, containing between 2 and 48 mosquitoes per pool (Table S3). In total, 13 of the 497 mosquito pools were found to be PCR-positive; the positive samples were mainly distributed in the Chongming, Jiading, and Pudong areas. There were three positive pools of *Culex tritaeniorhynchus* in Chongming and Pudong, respectively, five positive pools of *Culex tritaeniorhynchus* and two positive pools of *Anopheles sinensis* in Jiading, and no positives were detected in the Fengxian and Jinshan areas (Figure 1 and Table 3).

The PCR-positive samples were inoculated into BHK-21 cells and subjected to five blind passages, and the findings revealed that six samples exhibited CPEs. Three strains of JEV, denoted as SH22-M1, SH22-M9, and SH22-M14, were isolated from *C. tritaeniorhynchus* mosquitoes collected from pigsties. For the first time, two strains of JEV belonging to GI, denoted as SH22-M5 and SH22-M41, were isolated from *C. tritaeniorhynchus* mosquitoes from sheep sties, while one strain, SH22-M52, was isolated from Ae. Sinensis (Table 3 and Table 4).

### 3.3. MIR of JEV in Mosquitoes

Among the mosquito samples collected from the pig and sheep sties in the Chongming, Jiading, and Pudong Districts of Shanghai, 13 pools were positive for JEV, with a MIR of 0.8/1000. The MIR of JEV isolated from *C. tritaeniorhynchus* mosquitoes collected from the pigsties in Chongming was 0.95/1000. The MIR of JEV isolated from *C. tritaeniorhynchus* and *An*. *sinensis* collected from the sheep sties in Jiading was 0.62/1000 and 3.37/1000, respectively, while that of the JEV strains isolated from *C. tritaeniorhynchus* collected from the pigsties in Jiading was 0.53/1000. The MIR of JEV isolated from *C. tritaeniorhynchus* collected from the pigsties in Pudong was 0.72/1000 (Table 5).

### 3.4. Molecular Characterization and Phylogenetic Analysis of JEV

**Figure 2:**
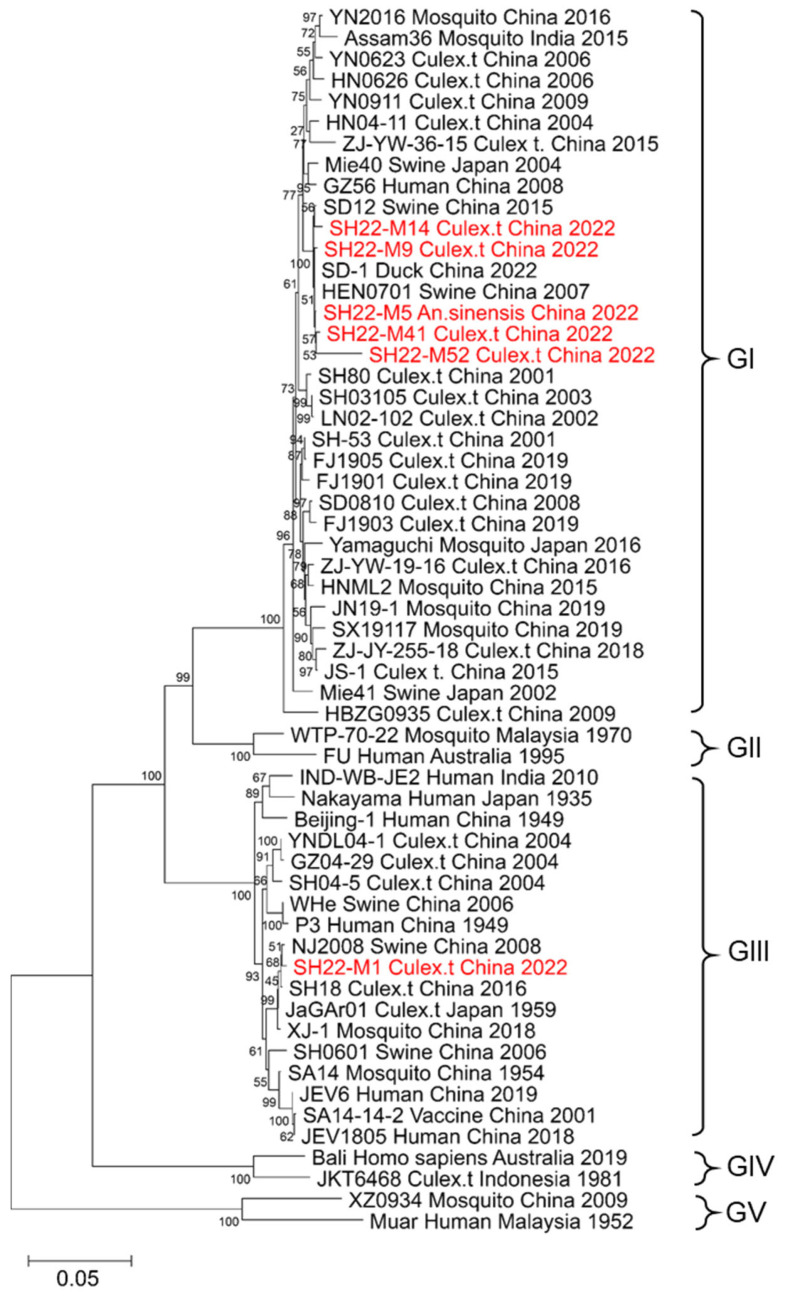
Phylogenetic analysis of JEV strains. The JEV strains isolated from the mosquitoes collected in this study were compared to representative JEV strains based on the nucleotide sequences of the E gene. The JEV sequences identified in this study are highlighted in red.

In order to elucidate the molecular genetic characteristics of the JEV isolates obtained in the present study, the reference sequences of the E gene of JEV retrieved from GenBank were subjected to multiple sequence alignment and phylogenetic analyses using MEGA X software. The phylogenetic trees were constructed using the neighbor-joining method, and the bootstrap values were derived from 1000 iterations. Genetic evolution analysis revealed that five of the six strains of JEV (SH22-M5, SH22-M9, SH22-M14, SH22-M41, and SH22-M52) belonged to the GI, while one strain (SH22-M1) was classified as GIII. The SH22-M9 strain was most closely related to the SD1 strain, with a sequence homology of 99.87%, while SH22-M14 shared the highest sequence homology of 99.53% with SD12. The SH22-M5, SH22-M41, and SH22-M52 strains were most closely related to the HEN07011 strain, with shared sequence homologies of 99.73–99.93%. SH22-M1 was most closely related to the SH18 strain, with a sequence homology of 99.8% (Figure 2). The first JEV strain found in Shanghai was isolated from a human brain in 1987 and classified as GIII, while the JEV isolates from Shanghai in 2001 and 2016 were GI. Although increasing evidence suggests that GI is replacing GIII as the predominant genotype in certain regions, GIII continues to persist along with GI in Shanghai.

### 3.5. Variations in the Amino Acid Residues of the JEV E Protein

The E protein of JEV is a major structural protein that contains 500 amino acids. It plays a crucial role in virulence, pathogenesis, virus binding to cellular receptors, and protective immunity against JEV [25,26,27]. The SH22-M1 strain of GIII, which was newly isolated from Shanghai in this study, differed from the SA14-14-2 strain used in live attenuated vaccines with respect to the following residues: E107 (F→L), E138 (K→E), E176 (V→I), E244 (G→E), E264 (H→Q), and E315 (V→A). The SH22-M1 isolate did not differ from the Indian isolate (IND-WB-JE2) at amino acid sites E107, E129, E138, E176, E222, E244, E264, E315, and E366 (Table 6). However, compared to the isolates from the surrounding Shanghai provinces of Fujian (FJ1901, FJ1905), Zhejiang (ZJ-JY-255-18, ZJ-YW-36-15), and Shandong (JN19-1), the key sites affecting virulence and immunogenicity in the SH22-M5, SH22-M9, SH22-M14, SH22-M41, and SH22-M52 strains, included E107 [28], E138 [29], E176 [30], and E244 [31]. The SH22-M5, SH22-M9, SH22-M14, SH22-M41, and SH22-M52 strains did not differ from the Japanese (Yamaguchi) and Indian (Assam36) isolates at the amino acid sites E107, E138, E176, and E244 associated with virulence and pathogenicity. Genotype I isolates (SH22-M5, SH22-M9, SH22-M14, SH22-M41, and SH22-M52) were compared to the Japanese isolate Yamaguchi, and the E366 amino acid site was identified by T→S (Table 6).

## 4. Discussion

JEV is the primary cause of viral encephalitis in humans and continues to spread extensively in South, East, and Southeast Asia, as well as Australasia [32]. In recent years, GI has been replacing GIII as the predominant genotype in the historical epidemic regions in Asia [33]. Genotype V (GV) emerged in Korea in 2010 [34], while an outbreak of genotype IV (GIV) was reported in mainland Australia in 2021 [35]. JEV is therefore considered to be an emerging and reemerging pathogen. The first JEV strain was discovered in 1987 in Shanghai following isolation from human brain tissue and was classified as GIII [5,36]. In contrast, the JEV strain belonging to GI was first isolated from *C. tritaeniorhynchus* in Shanghai in 2001 [37]. GI and GIII have been alternately detected between 2003 and 2008, indicating that both these genotypes coexist in circulation within the region [36]. GI was subsequently isolated from *C. tritaeniorhynchus* and *C. pipiens* mosquitoes in Shanghai in 2016 [5]. To the best of our knowledge, the surveillance of JEV vectors and mosquito-borne viruses has been limited in Shanghai. The present study investigated JEV infections in mosquitoes collected from pig and sheep enclosures in Shanghai in 2022. A total of 24,073 female mosquitoes were collected, belonging to seven species of four genera. *C. tritaeniorhynchus* was identified as the predominant species (87.09%, 20,965/24,073), followed by *C. bitaeniorhynchus* (4.73%, 1139/24,073), *An. sinensis* (4.13%, 995/24,073), and *Ae. albopictus* (3.54%, 853/24,073). The majority of the JEV-positive pools were isolated from *C. tritaeniorhynchus*, indicating that it is a major vector for the transmission of the virus [5]. The findings further revealed that *An. sinensis* also contributes to the circulation of JEV in Shanghai.

The characteristics of the genetic evolution of the JEV isolates were determined by sequencing the nucleotide sequences of the E gene of six strains of JEV. Genetic evolutionary analysis indicated that the SH22-M1 strain belonged to GIII, while SH22-M5, SH22-M9, SH22-M14, SH22-M41, and SH22-M52 were classified as GI. The sequence homology was highest between the SH22-M9 and SD-1 strains (99.87%) and between the SH22-M14 and SD12 strains (99.53%). The SH22-M5, SH22-M41, and SH22-M52 strains shared the highest sequence homology with the HEN07011 strain (99.73–99.93%). The SH22-M1 strain was found to be most closely related to the SH18 strain, with a sequence homology of 99.8%. Previous studies in our laboratory have shown that the JEV predominantly endemic in the mosquitoes and pig sera of the pig farms in Fujian Province, China, is of the GI type [18]. RT-PCR analysis of 805 mosquito pools collected in Zhejiang Province found that 69 pools were JEV-positive, with a positivity rate of 8.6%. E Genetic evolutionary analysis of the genes showed that the strains isolated from 2007 to 2014 were of the GI type, whereas the strains isolated from 1982 to 1983 were of the GIII type [16]. Of the 1495 mosquito pools collected from 2015 to 2018 in Zhejiang Province, 230 were found to be positive, with the highest JEV positivity rate of 16.33% found in *C. tritaeniorhynchus*. Phylogenetic analyses showed that 139 of the 230 positive pools were of the GI type. The Zhejiang strains can be divided into two genotypes: strains isolated in 1982 to 1983 are clustered into genotype GIII, while strains isolated from 2006 to 2018 are clustered into genotype GI [38]. Additionally, recent strains isolated from other regions both domestically and internationally also belong to genotype GI. Although increasing evidence suggests that GI is gradually replacing GIII as the predominant genotype, the present study revealed that GI and GIII continue to circulate widely among pigs in Henan, China [39]; both GI and GIII have been isolated from pigs in South China [27]. Recent studies have detected that GI is in circulation in Gorakhpur, Uttar Pradesh, and that GI and GIII are in cocirculation in West Bengal, India [40]. The monitoring of mosquitoes JEV in Shandong revealed that the minimum infection rates of *C. tritaeniorhynchus*, *C. pipiens*, and *Cq. Ochracea* were 5.29/1000, 1.60/1000, and 6.39/1000, respectively [41]. The mosquito populations in the pig farms and human habitats of these coastal cities (Fujian, Zhejiang, and Shandong) have a higher JEV positivity rate. Shanghai is closely adjacent to these cities, and as such, the results highlight the urgent need to enhance the surveillance of JEV infections in the mosquito vectors in Shanghai and closely monitor the epidemiological situation and trends in circulating genotypes. In addition, the present study isolated JEV strains for the first time from mosquitoes collected from sheep enclosures in Shanghai. The findings indicated that mosquito control measures should be reinforced not only in pig farms but also in other livestock environments (including sheep and cattle farms). The findings additionally highlight the necessity of enhancing JEV monitoring strategies. It is therefore recommended that livestock farming areas be separated from human habitation to mitigate the public health risk of JEV infections in humans.

It has been reported that the virulence of JEV is mainly determined by its structural proteins, especially the E protein, which plays a dominant role in JEV virulence [26]. The E protein is structurally divided into domains I (DI), II, and III [42]. The E138 amino acid is located in the “hinge” region at the junction of DI and DII. Mutations at this position alter the conformation and function of the protein, which, in turn, affects the virulence of GI and GIII strains. A previous study observed that mutations that introduce acidic or basic residues at position E138 alter the neurovirulence of JEV [29]. E107 is located in a highly conserved hairpin motif in DII and synergizes with residue 138 in determining the virulence of GIII strains. E123 is located in DII, and the mutation alters the critical pH required for the conformational change in the E protein prior to fusion with endosomal membranes [43]. E176, located in DI, also contributes to the neurotoxicity of GI strains in mice [30]. However, the SH22-M1 isolate did not differ from the Indian isolate (IND-WB-JE2) at amino acid sites E107, E138, E176, E244, E264, and E315. Genotype I isolates (SH22-M5, SH22-M9, SH22-M14, SH22-M41, and SH22-M52) did not differ from the Japanese (Yamaguchi) and Indian (Assam36) isolates, nor with the Fujian (FJ1901 and FJ1905), Zhejiang (ZJ-JY-255-18 and ZJ-YW-36-15), and Shandong (JN19-1) variants at the amino acid sites E107, E138, E176, and E244 associated with virulence and pathogenicity. Genotype I isolates (SH22-M5, SH22-M9, SH22-M14, SH22-M41, and SH22-M52) were compared with the Japanese isolate Yamaguchi, and the E366 amino acid site was identified by T→S [27]. The present study revealed that six residues—namely, F107L, K138E, V176I, G244E, H264Q, and V315A—in the SH22-M1-isolated strains of JEV belonging to GIII differed from those of the SA14-14-2 strain used in JEV vaccines. Therefore, the continuous monitoring of JEV is necessary for obtaining better insights into its genetic characteristics and enhancing the efficacy of JEV vaccines.

Shanghai is located on the eastern coast of China and has a subtropical monsoon climate [44], significantly influenced by the ocean. The ocean has a high heat capacity, allowing it to slowly absorb and release heat, which results in relatively small temperature fluctuations in Shanghai. Due to the continuous transfer of moisture from the ocean to the land, humidity is consistently high throughout the year, leading to substantial rainfall. This high-humidity environment provides favorable conditions for mosquito breeding [45], thereby promoting the outbreak and transmission of the Japanese encephalitis virus. It has been reported that weather factors, including temperature and precipitation, drive the transmission of Japanese encephalitis by affecting the mosquito lifecycle, including the development time of immature mosquito stages and mosquito density [45,46]. Studies have found that higher temperatures accelerate virus replication and increase the speed of transmission [47]. The transmission of JEV is related to agricultural activities and involves the mosquito transmission cycle in rice fields and the interaction between mosquitoes and humans in farmlands. Clearly, rice fields are the preferred breeding grounds for mosquitoes, thus providing an important transmission site for mosquito vectors [48,49]. In summary, improving the sanitation of farms, regularly controlling mosquitoes, and monitoring JEV, as well as adjusting irrigation methods to avoid water accumulation in rice fields, can significantly reduce the risk of JEV transmission, thereby improving the health of both humans and animals.

Currently, there are four different types of JE vaccines available for humans around the world, including mouse brain-derived inactivated, cell culture-derived live-attenuated, cell culture-derived inactivated, and genetically engineered live-attenuated chimeric vaccines [50]. The first licensed JE vaccine was an inactivated mouse brain-derived vaccine based on the Nakayama strain, which was highly immunogenic but had drawbacks such as high production costs and vaccine-induced side effects [50]. Due to the availability of newer cell culture-derived JE vaccines, the production of JE-VAX ceased in 2006, and the remaining stock expired in 2011 [51,52]. SA14-14-2 is the most widely used JE vaccine in JE endemic areas. Since 2007, China has included the JE vaccine in the National Immunization Program, providing preventive vaccination for children of an appropriate age (1–4 years). There was a significant reduction in morbidity after the inclusion of the JE vaccine in the Expanded Programme on Immunization (4.072/100,000 during 1970–2007 and 0.122/100,000 during 2008–2020) [53]. As of 2020, the vaccination rate of JEV in China has reached 99.25% [38]. These control measures have led to changes in the epidemiological characteristics of JE in China—in particular, a decrease in the number of cases and a change in the age structure of the affected population. Children are a high-risk group and a priority for immunization. The significant reduction in the incidence rate among high-risk groups confirms the effectiveness of vaccination [54,55]. Shanghai is located in Eastern China and, as one of the country’s major economic centers, has frequent trade exports. It is a key site on the East Asia–Australasia bird migration route, hosting a diverse population of migratory birds. This makes it a potential high-risk zone for the propagation of mosquito-borne viruses. Therefore, an increased monitoring of Japanese encephalitis virus in mosquitoes and the promotion and coverage of vaccination in the Shanghai region should be prioritized to reduce the risk of JE outbreaks.

## 5. Conclusions

This study identified the major mosquito species that serve as vectors of JEV in the Shanghai region. We isolated JEV from mosquitoes in sheep enclosures for the first time, and our findings revealed that GI and GIII are the predominant genotypes currently circulating in Shanghai. Therefore, monitoring the changes in JEV genotypes is crucial for developing effective control strategies. It is also necessary to reinforce the surveillance of mosquitoes in pig farms and enhance the monitoring of JEV infections in mosquitoes from pig and sheep enclosures.

## Figures and Tables

**Table 1:** Locations of the pig and sheep enclosures selected for collecting mosquito samples in Shanghai, China.

District	Date	Habitat	Farm ID	Temperatures	Longitude	Latitude
Chongming (CM)	2022.06.26	Sheepsties	CM1	21–29 °C	121.38649 °E	31.74885 °N
	2022.08.14	Pigsties	CM2	21–26 °C	121.28775 °E	31.74332 °N
Jiading (JD)	2022.07.17	Sheepsties	JD1	26–30 °C	121.2156 °E	31.439 °N
	2022.07.25	Sheepsties	JD2	26–30 °C	121.2056 °E	31.4198 °N
	2022.08.09	Pigsties	JD3	26–29 °C	121.1678 °E	30.8984 °N
Pudong (PD)	2022.07.11	Pigsties	PD1	27–36 °C	121.6775 °E	31.0343 °N
	2022.08.02	Pigsties	PD2	27–34 °C	121.7376 °E	30.9661 °N
Fengxian (FX)	2022.07.25	Pigsties	FX1	27–33 °C	121.538 °E	30.929 °N
Jinshan (JS)	2022.08.19	Pigsties	JS1	28–37 °C	121.2247 °E	30.8787 °N

**Table 2:** Distribution of the mosquito samples collected from Shanghai, China.

Mosquito Species	Collection Sites																		Total	
	Chongming				Jiading						Pudong				Fengxian		Jinshan			
	CM1		CM2		JD1		JD2		JD3		PD1		PD2		FX1		JS1			
	No.	%	No.	%	No.	%	No.	%	No.	%	No.	%	No.	%	No.	%	No.	%	No.	%
*C. tritaeniorhynchus*	2513	86.00	3173	88.93	4350	86.21	2136	78.96	1893	77.58	1293	92.56	2893	95.01	1152	90.64	1562	93.03	20,965	87.09
*C. bitaeniorhynchus*	152	5.20	74	2.07	271	5.37	184	6.8	315	12.91	28	2.00	36	1.18	21	1.65	58	3.45	1139	4.73
*An. sinensis*	23	0.79	128	3.59	325	6.44	269	9.94	156	6.39	21	1.50	41	1.35	13	1.02	19	1.13	995	4.13
*Ae. albopictus*	213	7.29	182	5.1	89	1.76	112	4.14	59	2.42	48	3.44	75	2.46	42	3.3	33	1.97	853	3.54
*C. pipiens pallens*	17	0.58	11	0.31	0	0.00	0	0	17	0.70	0	0.00	0	0.00	34	2.68	0	0	79	0.33
*C. pipiens* *quinquefasciatus*	4	0.14	0	0	0	0.00	0	0	0	0.00	7	0.50	0	0.00	9	0.71	0	0	20	0.08
*Ar. subalbatus*	0	0.00	0	0	11	0.22	4	0.15	0	0.00	0	0.00	0	0.00	0	0	7	0.42	22	0.09
Total	2922	100	3568	100	5046	100	2705	100	2440	100	1397	100	3045	100	1271	100	1679	100	24,073	100

**Table 3:** Identification of JEV-positive mosquito samples collected from pig and sheep enclosures in Shanghai, China.

Species	Number of Samples					Number of JEV-Positive Samples				
	CM	JD	PD	FX	JS	CM	JD	PD	FX	JS
*C. tritaeniorhynchus*	114	168	84	24	32	3 (1#)	5 (3#)	3 (1#)	0	0
*C. bitaeniorhynchus*	5	16	2	1	2	0	0	0	0	0
*An. sinensis*	4	15	2	1	1	0	2 (1#)	0	0	0
*Ae. albopictus*	8	6	3	1	1	0	0	0	0	0
*C. pipiens pallens*	1	1	0	1	0	0	0	0	0	0
*C. pipiens quinquefasciatus*	1	0	1	1	0	0	0	0	0	0
*Ar. subalbatus*	0	1	0	0	1	0	0	0	0	0

Number of positive samples: number of samples verified as positive by PCR; **#**: number of JEV strains isolated from the positive samples.

**Table 4:** Summary of mosquito-borne JEV isolates obtained from mosquito samples collected in Shanghai, China.

Isolates	Collection Sites	Habitat	Mosquito Species	Virus	Genotype
SH22-M1	Jiading	Pigsties	*C. tritaeniorhynchus*	JEV	GIII
SH22-M5	Jiading	Sheepsties	*An. sinensis*	JEV	GI
SH22-M9	Pudong	Pigsties	*C. tritaeniorhynchus*	JEV	GI
SH22-M14	Chongming	Pigsties	*C. tritaeniorhynchus*	JEV	GI
SH22-M41	Jiading	Sheepsties	*C. tritaeniorhynchus*	JEV	GI
SH22-M52	Jiading	Sheepsties	*C. tritaeniorhynchus*	JEV	GI

**Table 5:** MIR of JEV isolates obtained from the different mosquito samples collected in this study.

Collection Sites	Habitat	Mosquito Species	Number of Individuals	Number of Pools	Number of JEV Positive Pools	MIR (/1000)
Chongming	Pigsties	*C. tritaeniorhynchus*	3173	64	3	0.95
Jiading	Sheepsties	*C. tritaeniorhynchus*	6486	130	4	0.62
	Sheepsties	*An. sinensis*	594	12	2	3.37
	Pigsties	*C. tritaeniorhynchus*	1893	38	1	0.53
Pudong	Pigsties	*C. tritaeniorhynchus*	4186	84	3	0.72
Total			16,332	328	13	0.80

**Table 6:** Comparison of the amino acid residues at potential mutation sites of the E protein across different strains of JEV.

JEV Strain	Genotype	E107	E129	E138	E176	E222	E244	E264	E315	E366
SH22-M1	GIII	L	T	E	I	A	E	Q	A	A
SH22-M5	GI	L	M	E	I	S	E	Q	A	S
SH22-M9	GI	L	M	E	I	S	E	Q	A	S
SH22-M14	GI	L	M	E	I	S	E	Q	A	S
SH22-M41	GI	L	M	E	I	S	E	Q	A	S
SH22-M52	GI	L	M	E	I	S	E	Q	A	S
FJ1901	GI	L	M	E	I	S	E	Q	A	S
FJ1905	GI	L	M	E	I	S	E	Q	A	S
ZJ-JY-255-18	GI	L	M	E	I	S	E	Q	A	S
ZJ-YW-36-15	GI	L	M	E	I	S	E	Q	A	S
JN19-1	GI	L	M	E	I	S	E	Q	A	S
JS-1	GI	L	M	E	I	S	E	Q	A	S
SX19117	GI	L	M	E	I	S	E	Q	A	S
HEN0701	GI	L	M	E	I	S	E	Q	A	S
Yamaguchi	GI	L	M	E	I	S	E	Q	A	T
Assam36	GI	L	M	E	I	S	E	Q	A	S
IND-WB-JE2	GIII	L	T	E	I	A	E	Q	A	A
NJ2008	GIII	L	T	E	I	A	E	Q	A	A
SA14-14-2	GIII	F	T	K	V	A	G	H	V	A
FU	GII	L	T	E	I	S	E	Q	A	A
JKT6468	GIV	L	T	E	I	A	E	Q	A	S
Muar	GV	L	I	E	I	A	E	Q	A	A

## Data Availability

Data are contained within the article.

## Supplementary Materials

The following supporting information can be downloaded at https://www.mdpi.com/article/10.3390/ani14243653/s1: Table S1: Primers used for identification in this study; Table S2: Strains of Japanese encephalitis virus used in this study; Table S3: Distribution of sample pools with fewer than 50 mosquitoes sampled in Shanghai, China.
