# Supplementary material for: Genetic Characterization of Japanese Encephalitis Virus Isolates Circulating in Mosquitoes from Pig and Sheep Farms in Shanghai, China
Source: Animals (Basel). 2024 Dec 18;14(24):3653. doi: 10.3390/ani14243653 (PMC11672859; doi:10.3390/ani14243653)
Supplement: Supplementary file 1 [file animals-14-03653-s001.zip › Supplementary Table 1 and 2.pdf]

Genetic characterization of Japanese encephalitis viruses circulating in mosquitoes from pig or sheep farms in Shanghai , China

Hailong Zhang[1], Dan Li[2], Jiayang Zheng[2], Yan Zhang[2], Zongjie Li[2], Ke Liu[2], Beibei Li[2], Yafeng Qiu[2], Donghua Shao[2], Soesoe Wai[3], Jianchao Wei[2,], Zhiyong Ma[2,*], Juxiang Liu[1,*]

1 College of Veterinary Medicine, Hebei Agricultural University, Baoding 071000, China

2 Shanghai Veterinary Research Institute, Chinese Academy of Agricultural Sciences, Shanghai 200241, PR China

3 Department of Veterinary Public Health, University of Veterinary Science, Yezin 15013, Myanmar

Supplementary Table 1 Primers used for identification in this study

Primers	Primers sequence (5′ to 3′)	Amplify region	Size, base pairs
JEV-475-F	TTGGTCGCTCCGGCTTACA	Partial sequence of JEV E gene	475
JEV-475-R	GGTTTTCCGAGGTAGTGGTTC		
JEV-E-F1	TGYTGGTCGCTCCGGCTTA	Full-length sequence of JEV E gene	1500
JEV-R-F1	GATGTCAATGGCACATCCAGT		

Supplementary Table 2 Strains of Japanese encephalitis virus used in this study

Strain	Date	Region	Host	GenBank No.
SD12	2015	Shanghai, China	Swine	MH753127.1
YN2016	2016	Yunnan, China	Mosquito	MH385014.1
YN0911	2009	Yunnan, China	*Culex tritaeniorhynchus*	JF706267
HN0626	2006	Henan, China	*Culex tritaeniorhynchus*	JN381837.1
Mie 41	2002	Japan	Swine	AB241119
HEN0701	2007	Henan, China	Swine	FJ495189
YN0623	2006	Yunnan, China	*Culex tritaeniorhynchus*	JN381836.1
FJ1901	2019	Fujian, China	*Culex tritaeniorhynchus*	OQ181396
FJ1903	2019	Fujian, China	*Culex tritaeniorhynchus*	OQ181398
FJ1905	2019	Fujian, China	*Culex tritaeniorhynchus*	OQ181400
JN19-1	2019	Shandong, China	Mosquito	OM572538
SX19117	2019	Shaanxi, China	Mosquito	OM572540
ZJ-JY-255-18	2018	Zhejiang, China	*Culex tritaeniorhynchus*	MK095909.1
ZJ-YW-36-15	2015	Zhejiang, China	*Culex tritaeniorhynchus*	MK095789.1
JS-1	2015	Jiangsu, China	*Culex tritaeniorhynchus*	KX357114.1
LN02-102	2002	Liaoning, China	*Culex tritaeniorhynchus*	JF706278
GZ56	2008	Guizhou, China	Human	HM366552
ZJ-YW-19-16	2016	Zhengjiang, China	*Culex tritaeniorhynchus*	MK095863.1
HNML2	2015	Henan, China	Mosquito	KY927860.1
SD-1	2022	Shandong, China	Duck	OR711406
HBZG0935	2009	Hebei, China	*Culex tritaeniorhynchus*	JQ937334.1
HN04-11	2004	Henan, China	*Culex tritaeniorhynchus*	DQ404087
Mie 40	2004	Japan	Swine	AB241118
SD0810	2009	Shandong, China	*Culex tritaeniorhynchus*	JF706286
SH80	2001	Shanghai, China	*Culex tritaeniorhynchus*	JN381848
SH03105	2003	Shanghai, China	*Culex tritaeniorhynchus*	JN381846.1
SH53	2001	Shanghai, China	*Culex tritaeniorhynchus*	AY555757.1
FU	1995	Australia	Human	AF217620
WTP-70-22	1970	Malaysia	Mosquito	HQ223286
WHe	2006	Hubei, China	Swine	EF107523.1
Beijing-1	1949	Beijing, China	Human	L48961
P3	1949	Beijing, China	Human	U47032
Nakayama	1935	Nakayama, Japan	Human	AF112297
NJ 2008	2008	Nanjing, China	Swine	GQ918133
SA14	1954	China	Mosquito	U14163
SA14-14-2	2001	China	Vaccine	MK585066.1

GZ04-29	2004	Guizhou, China	*Culex tritaeniorhynchus*	DQ404111
SH18	2016	Shanghai, China	*Culex tritaeniorhynchus*	MH753132
XJ-1	2018	Xinjiang, China	*Culex pipiens*	MN529589.1
SH0601	2006	Shanghai, China	Swine	EF543861
JEV6	2019	Xinjiang,China	Human	MW766363.1
JEV1805	2018	China	Human	MN639770.1
YNDL04-1	2004	Yunnan, China	*Culex tritaeniorhynchus*	DQ404137
SH04-5	2004	Shanghai, China	*Culex tritaeniorhynchus*	DQ404106.1
JaGAr01	1959	Japan	*Culex tritaeniorhynchus*	AF069076
JKT6468	1981	Indonesia	*Culex tritaeniorhynchus*	AY184212
Bali	2019	Australia	Human	MT253731.1
Muar	1952	Malaysia	Human	HM596272
XZ0934	2009	Tibet, China	Mosquito	JF915894
Yamaguchi	2016	Japan	Mosquito	LC461957.1
IND-WB-JE2	2010	India	Human	JX072965
Assam36	2015	India	Mosquito	MT232844.1
